# Nanofiber self-consistent additive manufacturing process for 3D microfluidics

**DOI:** 10.1038/s41378-022-00439-2

**Published:** 2022-09-15

**Authors:** Bin Qiu, Xiaojun Chen, Feng Xu, Dongyang Wu, Yike Zhou, Wenchang Tu, Hang Jin, Gonghan He, Songyue Chen, Daoheng Sun

**Affiliations:** 1 Fujian Micro/Nano Manufacturing Engineering Technology Research Center, Xiamen University, Xiamen, 361102 China; 2 School of Mechanical and Electrical Engineering, Lingnan Normal University, Zhanjiang, 524000 China

**Keywords:** Electrical and electronic engineering, Microfluidics

## Abstract

3D microfluidic devices have emerged as powerful platforms for analytical chemistry, biomedical sensors, and microscale fluid manipulation. 3D printing technology, owing to its structural fabrication flexibility, has drawn extensive attention in the field of 3D microfluidics fabrication. However, the collapse of suspended structures and residues of sacrificial materials greatly restrict the application of this technology, especially for extremely narrow channel fabrication. In this paper, a 3D printing strategy named nanofiber self-consistent additive manufacturing (NSCAM) is proposed for integrated 3D microfluidic chip fabrication with porous nanofibers as supporting structures, which avoids the sacrificial layer release process. In the NSCAM process, electrospinning and electrohydrodynamic jet (E-jet) writing are alternately employed. The porous polyimide nanofiber mats formed by electrospinning are ingeniously applied as both supporting structures for the suspended layer and percolating media for liquid flow, while the polydimethylsiloxane E-jet writing ink printed on the nanofiber mats (named construction fluid in this paper) controllably permeates through the porous mats. After curing, the resultant construction fluid–nanofiber composites are formed as 3D channel walls. As a proof of concept, a microfluidic pressure-gain valve, which contains typical features of narrow channels and movable membranes, was fabricated, and the printed valve was totally closed under a control pressure of 45 kPa with a fast dynamic response of 52.6 ms, indicating the feasibility of NSCAM. Therefore, we believe NSCAM is a promising technique for manufacturing microdevices that include movable membrane cavities, pillar cavities, and porous scaffolds, showing broad applications in 3D microfluidics, soft robot drivers or sensors, and organ-on-a-chip systems.

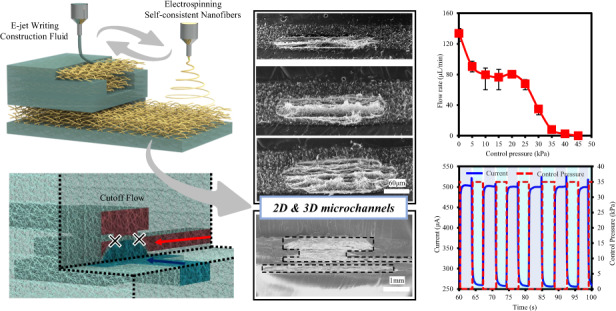

## Introduction

Over the past several decades, microfluidic chips have found extensive applications in various fields such as point-of-care testing^1^, environmental and food monitoring^2,3^, and biomedical engineering^4^ due to the integration of multiple functional units for diversified fluid control^5,6^. Compared with 2D structures, 3D microfluidic devices possess a higher density of functional structures (e.g., movable membranes^7–9^ and porous mats^10–13^), which enables complex fluid manipulation^14^ and multiplexed analytical tests^15^. Conventional 3D microfluidic chip fabrication is based on the microfabrication^16^ and chip bonding^17,18^ of multiple molded layers, which restricts the flexibility and diversity of microfluidic structures.

3D printing is a practical one-step technology that enables automatic, assembly free, and high-throughput fabrication of 3D microfluidic devices^19^ and mainly includes the photopolymerization method, multiple-jet modeling (MJM), and fused deposition modeling (FDM). Admittedly, photopolymerization provides a high-resolution channel formation method for microfluidic devices^20^, and FDM and MJM excel at constructing chips with multiple materials^21^. However, these 3D printing methods encounter a common problem of channel clogging^22,23^ when printing extremely narrow channels and T-shaped microchannels^24^. In addition, the 3D printing of suspended structures (e.g., movable membranes and cantilevers) faces the problem of collapse^20^ caused by liquid surface tension when removing sacrificial materials. Therefore, many printing–pause–printing strategies have been proposed to provide integrated fabrication schemes for 3D microfluidic channels^25^, which avoid sacrificial layer release processes. For example, Terry et al. printed polydimethylsiloxane (PDMS) lines by DIW on PMMA substrates as channel walls and then enclosed them with another layer of PMMA to form microchannels by chip bonding^26^. Andre et al. stacked a membrane on open channels as the supporting structure for the subsequent printing of channel cover layers^27^. Feng et al. and Dong-Woo et al. printed the expectant reagent in a channel by multiple nozzles before sealing the device^28,29^.

Herein, we propose a practical strategy named nanofiber self-consistent additive manufacturing (NSCAM) for direct 3D microfluidic fabrication by alternately employing electrospinning and an electrohydrodynamic jet (E-jet) writing. NSCAM is based on the self-consistent effect of porous nanofibers, which are prepared by electrospinning and utilized both as supporting layers for suspended structures and as percolating media for construction fluids. The E-jet ink, which is defined as construction fluid (CF), is printed on porous nanofibers and controllably permeates through the nanofibers, forming patterned 3D channel walls. The entire fabrication process can be realized automatically, without any sacrificial layer removal or bonding process. As a demonstration, a typical microfluidic pressure-gain valve^9^ with narrow 3D channels, cantilevers, and a movable membrane was fabricated and tested.

## Results and discussion

### Printing procedure of NSCAM for a 3D microfluidic chip

**Fig. 1:**
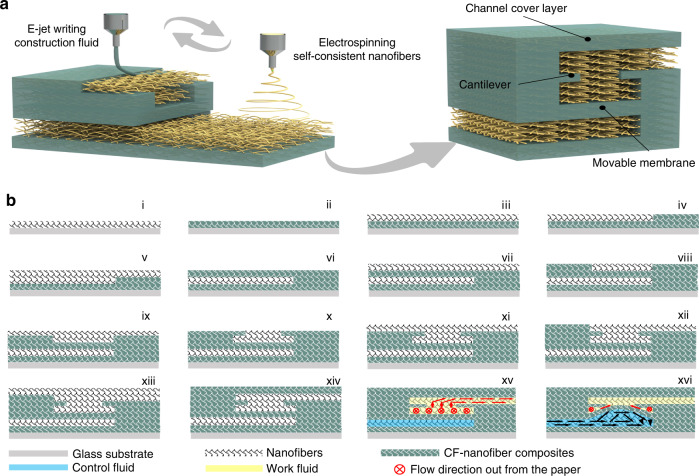
NSCAM procedures for 3D microfluidic devices. **a** 3D microfluidic channels fabricated by alternate electrospinning and E-jet writing. **b** The NSCAM process for a 3D microfluidic pressure-gain valve. i: electrospinning; ii: the E-jet ink is written on the nanofiber substrate and then permeates the porous membrane; iii, iv: the construction of the control channel layer by patterned CF penetration; v, vi: sealing the channel by controlling the CF vertical penetration distance; vii–xiv: the construction of the input channel layer, connecting layer, output channel and channel cover layer; xv–xvi: the principle of the 3D microfluidic pressure-gain valve

As shown in Fig. 1a, electrospinning and E-jet writing are alternately switched on in the printing procedure. The nanofibers formed by electrospinning are utilized as the porous substrate, and the E-jet ink is written on the nanofiber membrane and performs as CF. The spreading and penetration of the CF in the porous nanofibers are controlled by temperature, with an auxiliary heating substrate underneath. The heating substrate temperature is set to 90 °C, which enables a minimal penetration depth of ~45 μm. Figure 1b illustrates the NSCAM process in detail, taking 3D microfluidic valve printing as an example. The electrospun nanofibers, as the substrate for each layer, are utilized as the supporting material for the microchannels. In step (ii), the CF by E-jet writing penetrates the nanofibers thoroughly and forms the channel sidewalls after curing. In Step (vi), as shown in Fig. S1, channels are sealed by controlling the CF vertical penetration distance. The total channel height can be increased by layer-by-layer stacking by simply repeating the two processes. Further steps illustrate how a complex 3D valve structure is formed by NSCAM.

### 2D microchannels

**Fig. 2:**
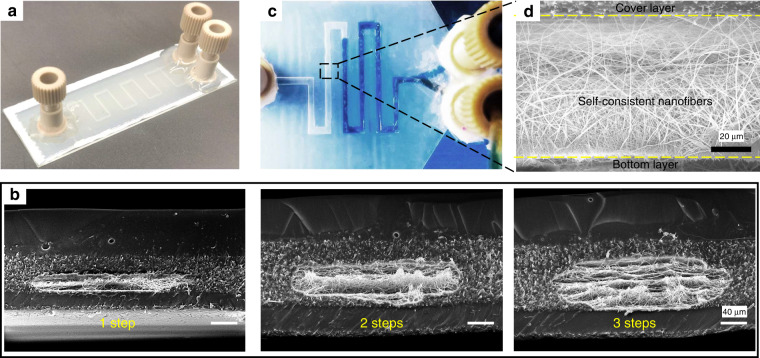
Microfluidic devices with self-consistent nanofibers in 2D channels. **a** A microfluidic chip with single-layered, long, thin channels; **b** microfluidic channels created by alternating electrospinning and E-jet writing for 1, 2, and 3 steps, respectively, during the preparation of the channel layer; **c** the flow continuity of self-consistent nanofiber-containing microchannels; **d** the vertical section of the microchannel with self-consistent nanofibers

**Fig. 3:**
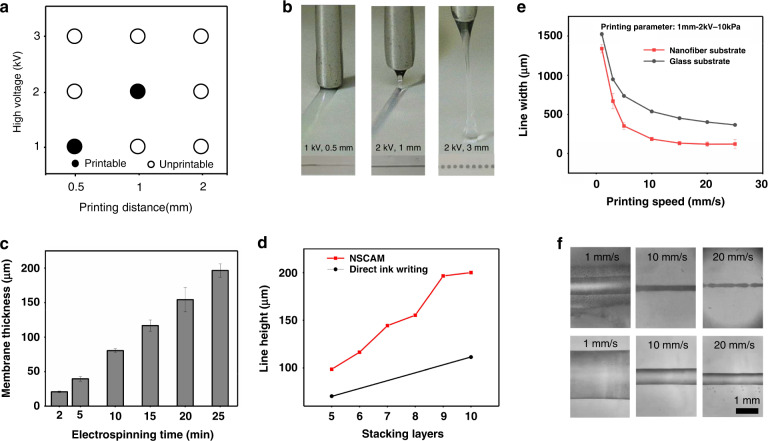
Electrospinning and E-jet writing characteristics in NSCAM process. **a** Printing performance at a variety of E-jet writing voltages and distances with printable (dark circles) and unprintable (light circles) combinations, **b** E-jet writing process under three different printing voltages and distance combinations; **c** the controlled thickness of the nanofiber membrane by electrospinning time; **d** the stacking height of printed lines by NSCAM and DIW; **e**, **f** the linewidth and morphology of printed lines on nanofiber mats and glass substrates

Electrospinning nanofibers possess consistency in the vertical direction. Thus, microchannels with different heights can be obtained by controlling the thickness of the electrospun nanofibers. Figure 2a shows single-layered long channels with a 263 μm wide and 56 μm high cross-section. Figure 2b presents microfluidic chips with different channel heights by alternating the electrospinning and E-jet writing steps. The thickness of the electrospun nanofibers in each step was kept at ~25 μm, according to Fig. 3c, to ensure thorough penetration of the CF into the nanofibers. By increasing the number of alternate steps, the channel height increased from 26 μm for 1 step to 51.23 μm for 2 steps and 76.54 μm for 3 steps. Figure 2c shows the injection of the working fluid (blue dye) in the channel. The working fluid flowed smoothly inside due to the porosity of the nanofibers. Figure 2d shows the consistent nanofibers in the channel, connecting the cover layer and bottom layer and supporting the suspended cover layer from collapse.

### NSCAM characteristics

In this study, PDMS was used as an E-jet ink, which is a widely used material in the field of microfluidics owing to its transparency, stretchability, and biocompatibility. Figure S2 shows the viscosity characteristics of the PDMS ink. As the proportion of hexane increases, the curing rate of the PDMS ink greatly slows down, guaranteeing ink stability during printing. Therefore, the E-jet ink in our study used a mixture of PDMS solution with 10%wt. hexane. To obtain printable E-jet writing parameters, orthogonal experiments were carried out, as shown in Fig. 3a, b and Fig. S3. The resulting optimal voltage parameter is 2 kV, and the optimal printing distance (namely, the distance between the E-jet writing nozzle and the slice position of printed structures) is 1 mm. The jet deflection intensifies with further lifting of the printing distance and increasing voltage.

Polyimide (PI) was used as the nanofiber material. It is a well-known polymer with excellent electrospinning characteristics, and its thermal stability makes it compatible with the PDMS printing process. As shown in Fig. 3c, the thickness of the electrospun nanofibers deposited on the PDMS substrate increases linearly as the electrospinning time increases from 2 to 25 min, with heights from 20 to 196 μm. To describe the uniformity in the electrospinning process, Fig. S7a–c present the thicknesses of the nanofiber membrane along the *x*-axis and *y*-axis, which are 147 ± 4.02 μm and 159.2 ± 1.68 μm, respectively. In Fig. S7d, e, the average diameter of the PI nanofibers is 234.3 nm, and the porosity of the electrospun membrane is 20.42%, which enables the percolation of the construction fluid and working fluid.

The morphology of printed lines is one of the most important characteristics of 3D printing technology. Figure 3d demonstrates the stacking heights of printed lines by NSCAM compared with direct ink writing (DIW) on a substrate without a nanofiber support. As the stacking number increases from 5 layers to 10 layers, the line height by NSCAM rises from 98.65 to 200.13 μm. In comparison, the stacking height by DIW increases from 70.48 μm for 5 layers to 111.42 μm for 10 layers, which indicates a much higher stacking efficiency for NSCAM. Figure 3e, f and Fig. S4 illustrate that the line width printed on the nanofiber substrate is narrower than that on the glass substrate, indicating a higher printing resolution for the nanofiber substrate. This can be attributed to vertical CF penetration into the porous nanofibers, which reduces its horizontal spreading.

**Fig. 4:**
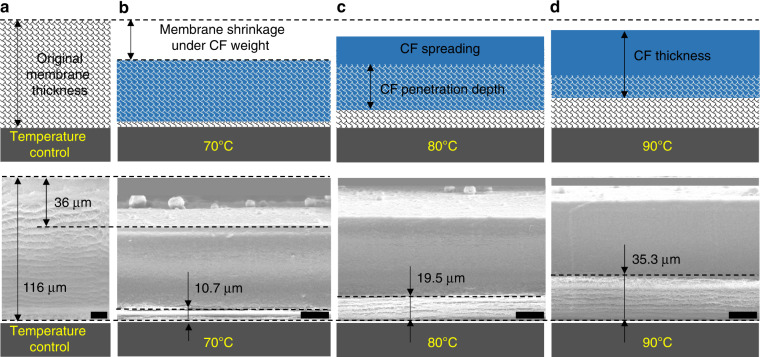
Vertical section of printed lines under different printing temperatures. **a** An electrospun membrane with original thickness of 116 μm. **b** Complete penetration of printed construction fluid into the porous membrane at the temperature of 70 °C. **c** Partial CF spreading because of the rapid CF solidification at the printing temperature of 80 °C. **d** The shorter CF penetration depth at the printing temperature of 90 °C (scale: 25 μm)

To precisely control CF penetration in the nanofiber layer, temperature, which greatly affects the curing time, is investigated as a critical factor for vertical penetration depth. A nanofiber membrane with a thickness of 116 μm was prepared by electrospinning, and CF ink was printed on the membrane at three different temperatures of 70, 80, and 90 °C. Figure 4 illustrates the temperature effect on the penetration depth. Compression of the nanofiber membrane under the CF weight was observed with an ~36 μm decrease in thickness, while the CF thickness (~70 μm) was determined by the CF volume and was fixed during the printing process. The remaining thickness of consistent nanofibers increases with printing temperature, from 10.7 μm at 70 °C to 19.5 μm at 80 °C and to 35.3 μm at 90 °C. To effectively use the electrospun nanofibers, a minimal penetration depth was chosen, which determined the thickness of a single nanofiber layer (~45 μm for 90 °C). An even higher temperature will reduce the printing stability; therefore, a temperature of 90 °C is used for the remaining experiments.

### Microfluidic pressure-gain valve

**Fig. 5:**
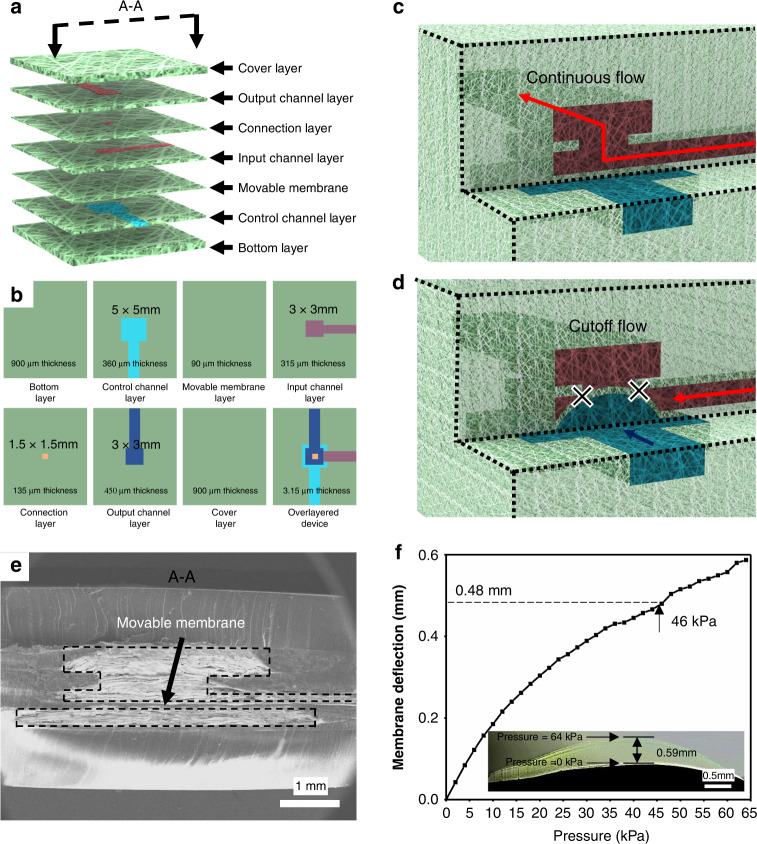
Structural characteristics of the printed microfluidic valve. **a** Schematic of the microfluidic pressure-gain valve; **b** the designed sizes of the valve; **c**, **d** switching principle of the microfluidic pressure-gain valve; **e** vertical section of the printed valve; **f** membrane deflection under air pressure in the control channel

To demonstrate the applicability of this method, a microfluidic pressure-gain valve was fabricated, as shown in Fig. 5. A microfluidic pressure-gain valve is a typical microdevice with 3D channels, cantilevers, and a movable membrane, which was proposed to realize efficient flow control^8^. The pressure-gain valve contains seven parts: (1) the bottom layer, (2) the control channel layer, (3) the movable membrane, (4) the input channel layer, (5) the connection layer, (6) the output channel layer, and (7) the cover layer, whose designed sizes are described in Fig. 5b. When air pressure was applied to the control channel, the movable membrane was deflected toward the connection layer. As the control pressure increases, the displacement reaches the maximum point where the membrane completely blocks the connection hole, cutting off the flow rate in the output channel layer. As seen in Fig. 5f, when the air pressure in the control channel increases from 0 kPa to 64 kPa, the maximum deflection point located in the center of the membrane reaches the maximum displacement of 590 μm. Figure 5e and Table S1 present the 3D structure and corresponding sizes of the printed valve. The height and width of the control channel are approximately 350 μm and 5 mm, respectively, indicating a width/height ratio of 15.

**Fig. 6:**
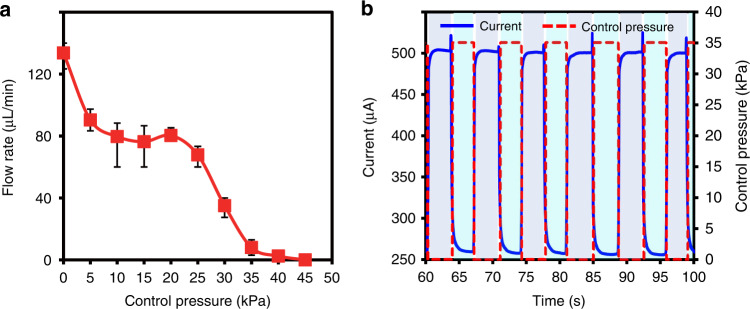
Functional characteristics of the printed microfluidic valve. **a** Static performance of the printed microfluidic device under different control pressures. **b** Dynamic response of the printed microfluidic device for multiple loading‒unloading circulations

Figure 6a shows the static performance of the printed microfluidic device. The working flow rate with an initial value of 135 μL/h decreases as the control pressure rises, which mainly results from the large compression of nanofibers in the inlet channel, and the cutoff pressure appears at 45 kPa due to the block of the connection layer. There is a refractory period between 10 and 20 kPa because of the finite compression of nanofibers, which favors stable pressure control for a fixed flow rate of ~80 μL/min.

To evaluate the dynamic performance of the valve, a testing system was established, as shown in Fig. S5. By loading a control pressure, the impedance of the working flow increases due to valve blocking; thus, the electric current passing through the whole circuit drops. Figure 6b shows the stable and repeatable response of the printed microfluidic valve. The current value dropped from 500 to 250 μA when the pressure rose from 0 to 35 kPa, and a delay of ~52.6 ms between the pressure loading and current change was observed. Multiple loading–unloading circulations with a frequency of 0.15 Hz were conducted and showed no structural failure for 100 repetitions. Therefore, the dynamic performance of the valve in this work is consistent with counterparts in previous literature^30,31^, suggesting the feasibility of the NSCAM strategy.

## Conclusions

In this paper, we proposed a highly efficient additive manufacturing strategy for 3D microfluidic fabrication, which can manufacture complex 3D microfluidic devices without a sacrificial layer release process. The porous nanofibers prepared by electrospinning were ingeniously utilized as the supporting structures and percolating media, and E-jet ink was printed into the nanofibers to construct the channel walls. By optimizing the process parameters, a horizontal resolution of 120 μm and a vertical resolution of 45 μm were obtained. As a proof of concept, a microfluidic pressure-gain valve was fabricated, demonstrating the feasibility of this strategy for manufacturing 3D microfluidic devices with multiple materials, dimensions, and components. This work provides a novel fabrication strategy for microdevices containing movable membrane cavities, pillar cavities, and porous scaffolds, showing a promising future for applications in soft robotics^32^, biomedical engineering^33^, and analytical chemistry^34^.

## Materials and methods

### Materials

A mixture of polyimide (PI) and dimethylacetamide (DMAc) (SG120 L, Hangzhou Surmount Science & Technology, Hangzhou, China) with a dynamic viscosity of 20,000 cps was used as the electrospinning solution without extra treatment after purchase. E-jet ink (or CF) was prepared by blending PDMS (Sylgard 184, Dow Corning) prepolymer, curing agent, and hexanes (Shanghai Macklin Biochemical Co., Ltd) at a ratio of 90:9:11. The mixture was stirred manually for 3 min, followed by degassing in a vacuum oven (DZF-6012, Shanghai Yiheng Scientific Instrument Co., Ltd.) for 5 min. The E-jet ink was used within 6 h after mixing to avoid obvious rheological property changes. The working fluid used in Fig. 2c was prepared by diluting food dye solution (Shandong Kaibei Food Co., Ltd.) with deionized water (obtained from Master-Q30UT, Shanghai Hetai Instrument Co., Ltd.) with a ratio of 100:1. The working fluid used in Fig. 6 was obtained by dissolving KCl powder (Xilong Chemical Co., Ltd.) in deionized water for a concentration of 10 mM.

### Custom-made 3D printing system

A hybrid 3D printing system was established, as shown in Fig. S6, enabling electrospinning and E-jet writing in the NSCAM process. The 3D printing system contains two sets of nozzle modules mounted on the *Z*1-axis and *Z*2-axis for electrospinning and E-jet writing, respectively. A direct current (DC) high-voltage power supply (DW-P403-1AC, Dongwen) was used to provide an electric field between the nozzle and the collection platform during the electrospinning and E-jet writing process. The collection module, which contains an auxiliary heating substrate, was mounted on an orthogonal *X*–*Y* axis mobile stage (Googoltech, Shenzhen, China). An ITO-coated glass sheet (25 × 25 × 1 mm, Yiyang Southern China Xiangcheng Technology Co., Ltd.) was used as the printing substrate and was placed on the auxiliary heating plate. The anode of the DC high-voltage source was connected to the nozzle, and the cathode was connected to the auxiliary heating plate. The distance between the nozzle and the collection platform was adjusted by moving the *Z*-axis. The electrospinning process parameters were set to a voltage of 9 kV, a distance of 10 cm, and an extruded pressure of 135 kPa, according to previous literature^35^. All experiments were conducted at room temperature, 1-atmosphere pressure, and 50% relative humidity.

### Material and structural characterization

The viscosity of the E-jet ink was measured by a rotational viscometer (ViscoQC 100 L, Anton Paar Trading Co., Ltd., Shanghai). The morphology of the printed lines was observed with an optical microscope (Chongqing Aote Optical Instrument Co., Ltd.), while the PDMS-nanofiber composites and the vertical section of printed structures were observed with scanning electron microscopy (SEM, JSM-IT500, JEOL Ltd.). A profilometer (DektakXT, U.S.A) was used to measure the thickness of nanofiber membranes and the profile of printed PDMS lines. The jet status of electrospinning and E-jet writing and the deformation of the membrane were recorded by a CMOS industrial camera (Huawang Image Technology Co., Ltd.). The precision syringe pump (Harvard, U.S.A.) was used to drive the fluid. The fluid rate was measured by a flow gauge (FLOW-EZ, FLEUIGENT). The air pressure was controlled by a PACE modular pressure controller (PACE6000, Druck). To obtain the dynamic performance of the microfluidic pressure-gain valve, a voltage source (KXN-155DM, Zhaoxin) was used to supply a voltage of 12 V, and the electric current was measured by a digital multimeter (Agilent 34410A, USA).

## Supplementary information

Supplementary Information
