# Supplementary material for: Nanofiber self-consistent additive manufacturing process for 3D microfluidics
Source: Microsyst Nanoeng. 2022 Sep 15;8:102. doi: 10.1038/s41378-022-00439-2 (PMC9477890; doi:10.1038/s41378-022-00439-2)
Supplement: Supplementary file 1 — Supplementary Information [file 41378_2022_439_MOESM1_ESM.docx]

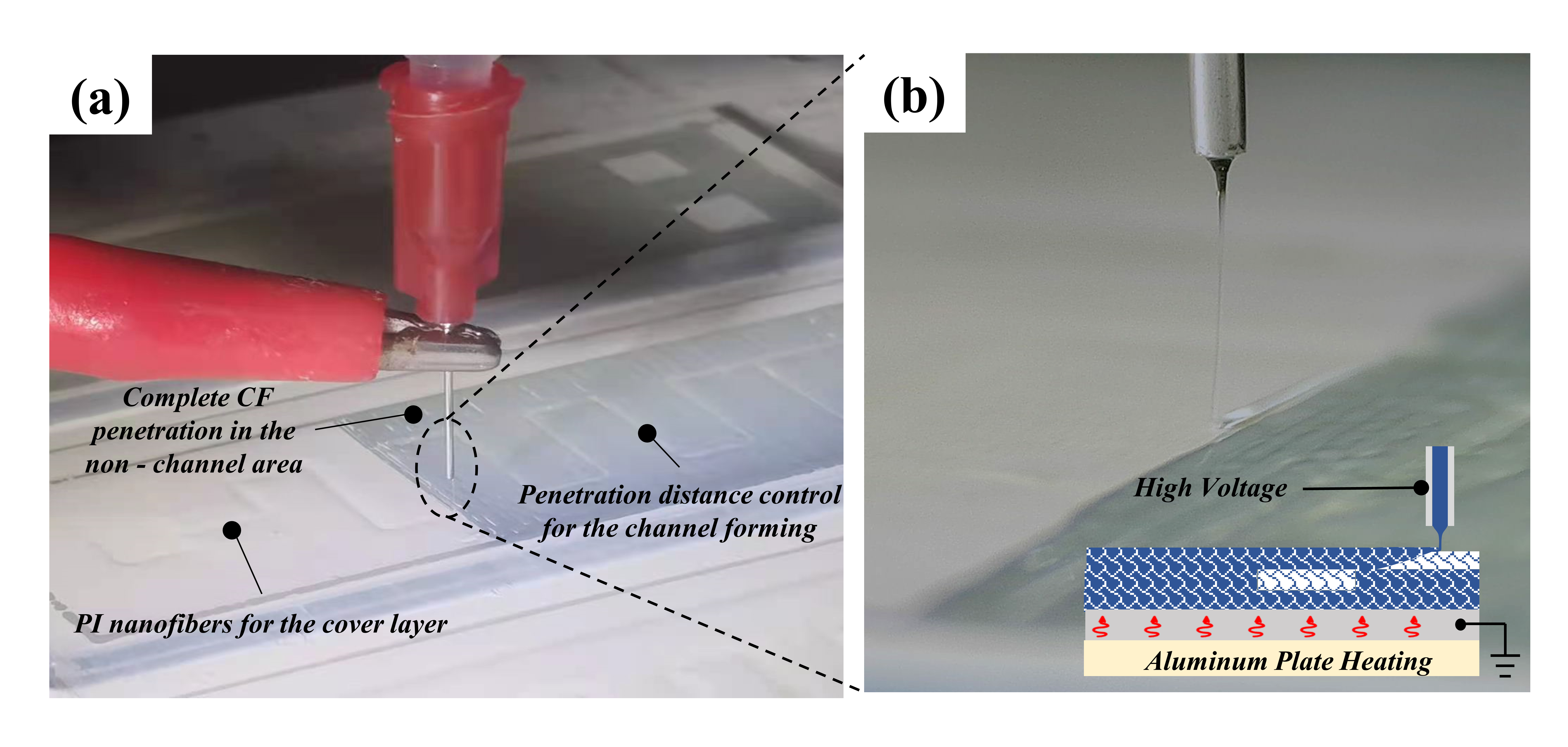


**Figure S1.** The E-jet process for single-layered channels. (a) the printing scene for the cover layer of channels. (b) the jet deflection at 3 mm printing distance.





**Figure S2.** Viscosity of PDMS ink diluted by hexane in different ratios.


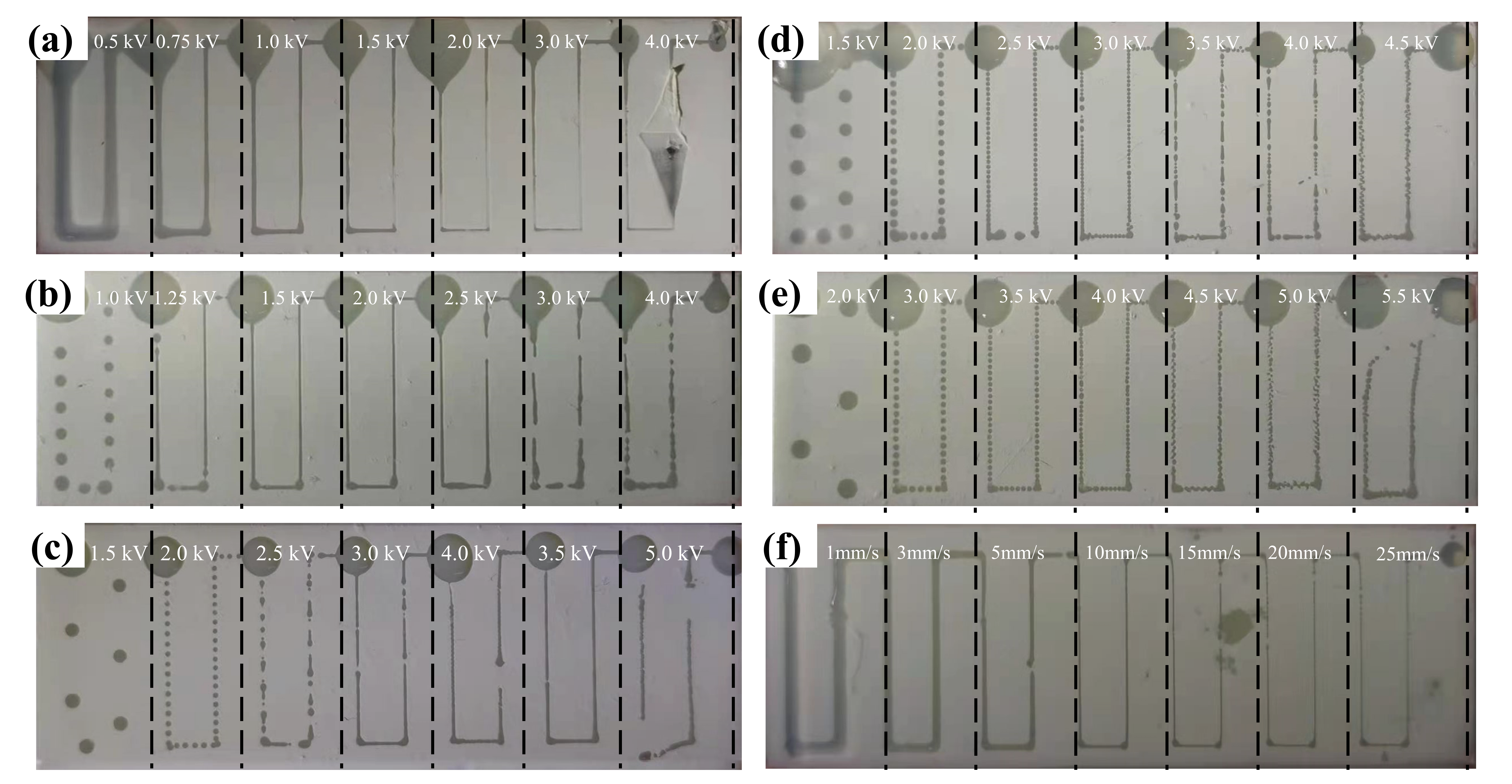


**Figure S3.** Voltage, distance and speed of the E-jet writing impact on the printing stability. (a-e) Morphology of the printed line with different voltage under the distance of 0.5 mm, 1 mm, 2 mm, 3 mm, 4 mm respectively; (f) The line width decreases as printing speed ranges from 1 mm/s to 25 mm/s with 1 mm printing distance and 2 kV voltage.


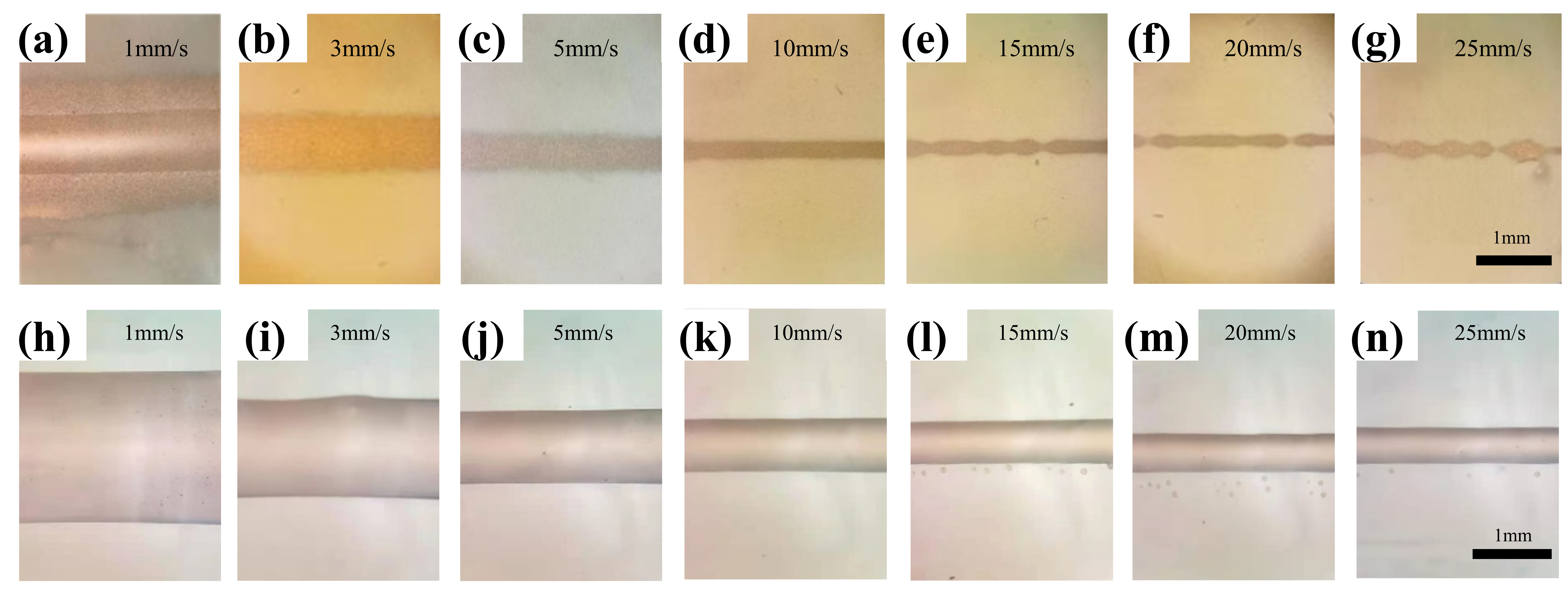


**Figure S4.** Morphology of printed lines under different printing speed with 1 mm printing distance and 2 kV voltage. (a-g) nanofiber substrate. (h-n) glass substrate.


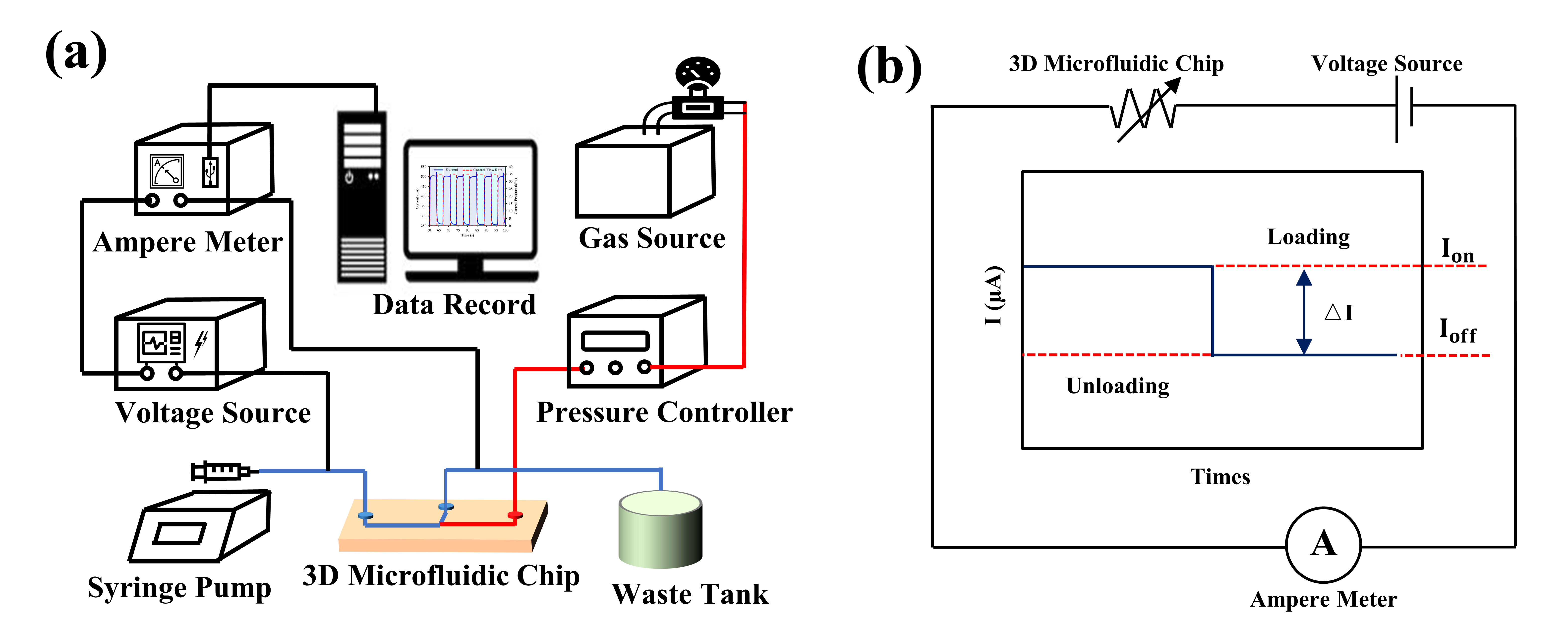


**Figure S5.** Microfluidic valve dynamic performance test. (a) system and (b) principle


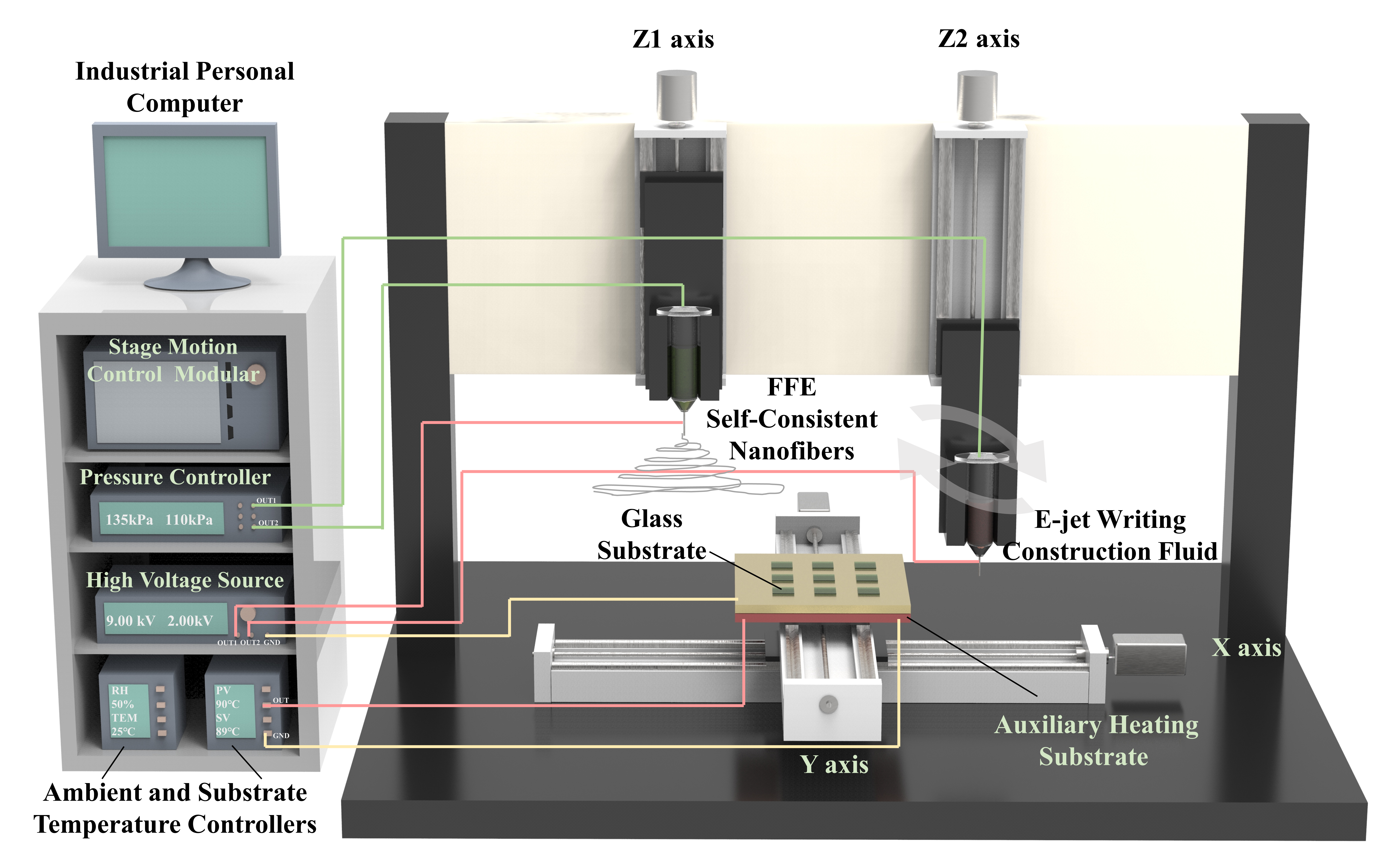


**Figure S6.** Custom-made hybrid EHD printing system enabling the electrospinning and electrohydrodynamic jet (E-jet) writing.


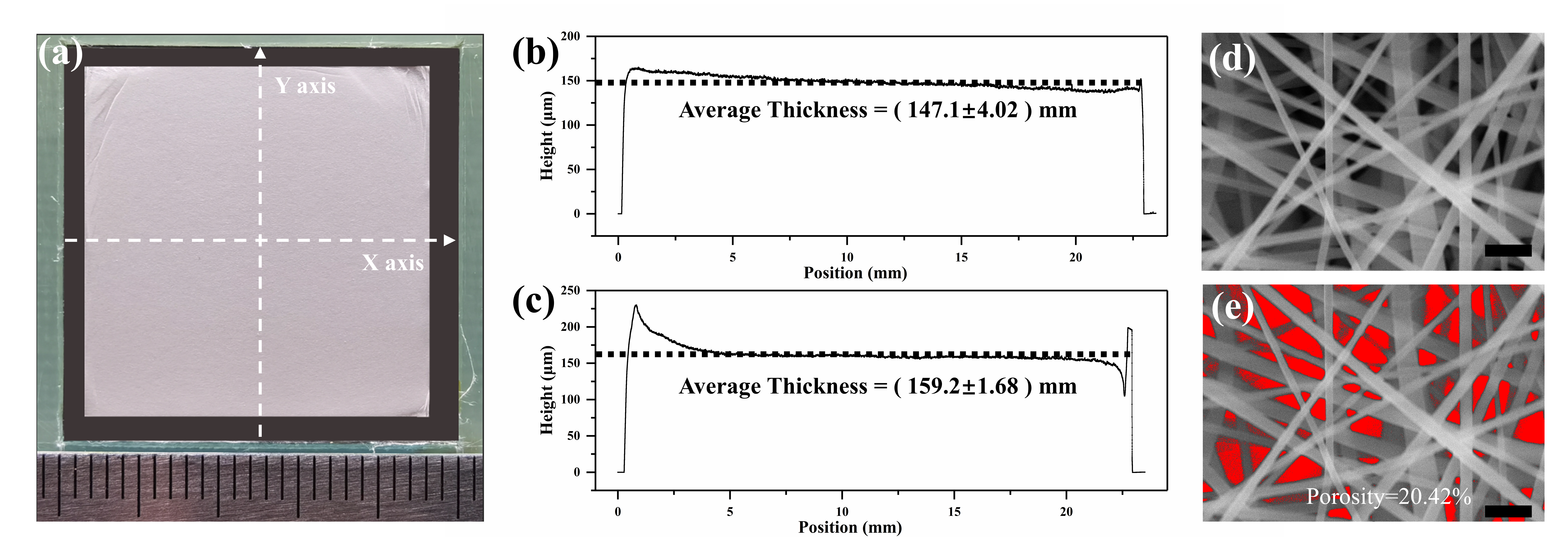


**Figure S7.** Thickness characteristics of electrospun membrane. (a) the orthogonal scan direction by profilometer for the thickness measurement of membrane; (b-c) the thickness distribution along the x and y axis respectively; (d) the micromorphology of electrospun PI nanofibers (scale: 1 μm); (e) the porosity calculation of nanofiber membrane.

**Table S1.** The structure size of printed microfluidic valve

| Structures | Length/Width (mm) | Thickness (μm) |
| --- | --- | --- |
| Control channel | 5.08  3 | 344 |
| Membrane | / | 92 |
| Input layer | 3.58 | 290 |
| Connection layer | 1.77 | 134 |
| Output layer | 3.39 | 427 |
